# The prevalence, determinants, and mechanisms of traditional Chinese medicine service utilization among healthcare professionals: a study based on the Andersen Behavioral Model

**DOI:** 10.3389/fpubh.2026.1827392

**Published:** 2026-05-13

**Authors:** Jie Huang, Zhouxiang Li, Yulei Chen, Ruyu Li, Xiyue Wang, Xinru Kong, Yuexia Gao

**Affiliations:** 1 Department of Health Management, School of Public Health, Nantong University, Nantong, China; 2 Nantong Center for Disease Control and Prevention, Nantong, China; 3 Nantong Hospital of Traditional Chinese Medicine, Nantong, China; 4 The Second People’s Hospital of Tongzhou District, Nantong, China

**Keywords:** Andersen’s Behavioral Model, loyalty, perceived value, satisfaction, traditional Chinese medicine service utilization

## Abstract

**Background:**

As a cornerstone of China’s healthcare system, Traditional Chinese Medicine (TCM) contributed significantly to both public health outcomes and cultural preservation. Although perceived value was known to shape healthcare decisions, its specific impact on TCM utilization among medical professionals remained unclear. This study employed the Andersen Behavioral Model to examine how predisposing, enabling, and need factors influenced TCM utilization among healthcare workers, with a focus on the mediating roles of usage intention and loyalty.

**Methods:**

A cross-sectional survey was administered to 1,483 healthcare professionals. We collected data on demographic profiles, TCM-related perceptions (perceived value, satisfaction, intention, and loyalty), and actual service usage over the preceding year. To identify predictors of utilization and test the hypothesized mediation pathways, we performed multiple logistic regression analyses and utilized the Model 4 macro in the SPSS PROCESS plugin.

**Results:**

The survey revealed that 49.09% of participants had utilized TCM services, with massage and manual therapies being the predominant modalities. Logistic regression demonstrated that sex, officially budgeted post, physical pain or discomfort, and chronic conditions significantly predicted utilization. Perceived value (OR = 1.037, *p* < 0.001) and satisfaction (OR = 1.149, *p* < 0.001) emerged as strong positive drivers. Mediation analysis confirmed that utilization intention mediated the relationship between perceived value and actual behavior (indirect effect = 0.023, 95%CI: 0.007–0.041), while loyalty mediated the link between satisfaction and utilization (indirect effect = 0.056, 95%CI: 0.009–0.101).

**Conclusion:**

TCM utilization among healthcare professionals was driven by a combination of predisposing factors (sex, officially budgeted post, perceived value, satisfaction) and need factors (chronic disease status, physical pain or discomfort) in the Andersen Model. The mediating effects of intention and loyalty highlighted the importance of psychological factors in service adoption. To boost TCM engagement, interventions should be implemented based on healthcare professionals’ clinical needs and educational level, while also fostering stronger loyalty and usage intention. These results offered actionable insights for optimizing TCM service delivery within the medical community.

## 1 Introduction

Traditional Chinese Medicine (TCM) stands as a distinctive health resource in China (1), serving a crucial dual function in both safeguarding public health and preserving national cultural heritage (2). Clinically, it is extensively utilized for chronic disease management and rehabilitation, particularly favored by older adults for its cost-effectiveness, minimal side effects, and efficacy in addressing long-term conditions (3). While TCM has garnered international recognition as a complementary health service (4–6) and is increasingly valued for its holistic and preventive benefits (1), its application within China remains predominantly treatment-oriented. This limits its integration into preventive care and rehabilitation (7), a scenario that belies its inherent advantages. Furthermore, domestic utilization is uneven, characterized by significant regional disparities and underutilization in certain areas (8). This is partly attributed to inadequate resource allocation, but a more critical factor is the insufficient knowledge of TCM among healthcare professionals themselves. As key stakeholders, their utilization behavior not only reflects their professional recognition of TCM but also potentially influences patient decisions. Consequently, investigating the utilization patterns of healthcare professionals and their determinants is essential. Such research can facilitate the optimization of TCM integration into the national healthcare system and maximize its public health value.

Scholarly attention toward TCM service utilization has intensified in recent years, with numerous studies identifying a spectrum of influencing factors. Existing literature has established demographic characteristics (9), socioeconomic status (10), and healthcare-related variables as key determinants of utilization (2). For example, consistent positive correlations have been observed between higher educational attainment, comprehensive medical insurance coverage, and the use of TCM services. However, prior research exhibits two significant limitations (11). Specifically, the focus has largely remained on older populations or the general public, with minimal attention paid to healthcare professionals. Given their unique professional cognition and distinct access to TCM resources, the utilization behaviors of this group necessitate specific, targeted investigation. Furthermore, most studies focus on the direct association between demographic or socioeconomic factors and TCM utilization. Relatively few studies examine the role of intermediate mechanisms such as perceived value. Even fewer studies integrate these factors into a systematic theoretical framework. This fragmentation makes it difficult to clarify the hierarchical relationships between factors and the path through which they influence utilization. It also means we still do not fully understand how factors lead to TCM utilization behavior.

To address this gap, this study adopts the Andersen Behavioral Model of Health Services Use as its theoretical framework. Developed by Anderson and Aday from the University of Chicago in 1968, this model is one of the most classic and widely used frameworks for studying health service utilization globally (12, 13). It has also been effectively applied in Chinese healthcare research, such as analyzing general medical service utilization and primary care access (14, 15). The Andersen Model posits that health service utilization is determined by three hierarchical dimensions: Predisposing Characteristics, Enabling Resources, and Need Factors. Predisposing factors encompass demographic attributes (age, sex, marital status), occupational characteristics (type of hospital, occupation, officially budgeted post), and health cognition (perceived value, satisfaction), while enabling resources refer to socioeconomic resources (working years, professional title, education level) and service accessibility. Need factors are defined by health need, such as chronic condition, physical pain or discomfort, sleep quality, and self-rated health. The framework suggests a sequential pathway where Predisposing Characteristics influence Health Service Utilization indirectly, mediated by Enabling Resources and Need Factors (16–18). Given that TCM utilization among healthcare professionals constitutes a specific form of health service behavior, it aligns naturally with this analytical logic. Specifically, demographic traits may shape TCM cognition, professional credentials may dictate access conditions, and subjective evaluations may drive actual decision-making. Consequently, the Andersen Model offers a systematic framework for integrating these diverse influences. To address the model’s limitation in explaining the translation from attitudes to actions, this study further incorporates intermediate mechanisms into the analytical framework.

Against this backdrop, this study focuses on healthcare professionals as the primary research subject. By integrating the three dimensions of the Andersen Behavioral Model and incorporating intermediate mechanisms into the analytical framework, this research explores the determinants of TCM service utilization within this group. The study addresses three core objectives. First, it investigates the current prevalence of TCM utilization among healthcare professionals and identifies the most frequently employed modalities. Second, grounded in the Andersen Model, the study examines which factors, spanning predisposing, enabling, and need dimensions, exert a significant influence on utilization, while also assessing their relative importance. Finally, the research explores the mediating roles of utilization intention and loyalty within these causal pathways.

Through these inquiries, this study aims to systematically elucidate the current status and underlying mechanisms driving TCM service utilization among healthcare professionals. Theoretically, this study enriches the application of the Andersen Model in the research on TCM service utilization among healthcare professionals. It also helps clarify the specific path of how factors influence TCM service utilization behavior. Practically, the findings can provide references for understanding the current status of healthcare professionals’ TCM utilization. They also offer targeted suggestions for promoting the rational use of TCM services in the healthcare system.

## 2 Methods

### 2.1 Data sources

In May 2024, this study employed quota sampling and used a self-designed Questionnaire on the Utilization of Traditional Chinese Medicine Services to conduct face-to-face surveys with healthcare professionals.

The inclusion criteria were as follows: (1) Medical institutions encompassed comprehensive hospitals, traditional Chinese medicine hospitals, specialized hospitals, and township health centers. (2) Staff members comprised physicians, nurses, medical technicians, and administrative personnel. (3) Being 18 years of age or older; (4) Providing informed consent and voluntarily participating in the survey. The exclusion criteria were: (1) Incomplete submission of data. (2) Logical inconsistencies in the responses.

The sample size calculation formula for counting data in descriptive research is


$$n=\frac{u_{\alpha }^{2}\pi (1-\pi )}{\delta^{2}}$$

A significance level of *α* = 0.05 was adopted, with an allowable error of *δ* = 0.03. For the general population proportion (*π*), the utilization rate of TCM services was set at 50%, i.e., π = 50%. We determined the minimum sample size requirement to be approximately 1,067 households. To ensure a representative cross-section of healthcare settings, we allocated 580, 551, and 397 questionnaires to tertiary, secondary, and primary institutions, respectively. We ultimately collected 1,483 valid questionnaires, resulting in an effective response rate of 97.05%.

### 2.2 Measurement of variables

#### 2.2.1 Dependent variable

TCM service utilization served as the outcome variable, determined by participants’ self-reported usage within the preceding 12 months. Responses were dichotomized, where “no use” was coded as 0 and “use” as 1.

Additionally, a multiple-choice item was used to identify the specific modalities of TCM employed by the participants during this period. Options include acupuncture, tuina massage, moxibustion, external TCM therapies, Chinese herbal medicine, internal TCM therapies, modern auxiliary devices, surgery, health guidance, and others.”

#### 2.2.2 Independent variables

Independent variables were categorized into predisposing factors, enabling factors, and need factors in accordance with the Andersen Behavioral Model.

##### 2.2.2.1 Predisposing factors

Predisposing factors included demographic characteristics (age, sex, marital status), occupational characteristics (type of hospital, hospital level, occupation, officially budgeted post, working years), and health cognition factors (perceived value, satisfaction).

This study adopted Chen et al.’s perceived value scale (19), covering economic, functional, and trust value dimensions with 12 items across 5 indicators: functional, emotional, and trust values. Economic value includes 3 items (e.g., TCM services, medicines, overall cost-effectiveness); functional value comprises 3 items (e.g., satisfaction with efficacy, treatment process, costs); trust value involves 6 items (e.g., TCM competence, practitioners, service capacity, reputation). All items used a 1 to 5 rating scale, with each dimension’s score being the sum of its items. Perceived value was operationalized as a composite measure incorporating economic, functional, and trust dimensions, with higher aggregate scores reflecting more favorable perceptions. The scale demonstrated good internal consistency, achieving a Cronbach’s *α* coefficient of 0.822.

Satisfaction was evaluated using a 4-item scale developed through systematic literature review and expert validation. These items assessed participants’ views on treatment experiences, the practical value of TCM technology, service quality relative to Western medicine, and overall contentment. Responses were recorded on a 5-point Likert scale (1 = strongly disagree, 5 = strongly agree). With a Cronbach’s *α* of 0.906, the scale exhibited high reliability.

##### 2.2.2.2 Enabling factors

Enabling factors included professional title, educational level, and TCM service accessibility.

Accessibility was assessed using three items measuring travel time to the nearest TCM institution, appropriateness of distance, and adequacy of service coverage. All items were scored on a 5-point Likert scale ranging from 1 to 5, with higher aggregate scores indicating better accessibility to TCM services. The scale demonstrated acceptable reliability, with a Cronbach’s *α* of 0.688.

##### 2.2.2.3 Need factors

Need factors included chronic conditions, physical pain or discomfort, sleep quality, and self-rated health.

Physical pain or discomfort was measured by one item asking how often participants had been bothered by pain or physical discomfort over the past year. Responses were rated on a 5-point scale (1 = never, 5 = very often), with higher scores indicating more frequent and severe physical discomfort.

Sleep quality was evaluated using one item assessing overall sleep quality over the past year. Responses were recorded on a 5-point scale (1 = very poor, 5 = very good), where higher scores reflected better sleep quality.

Self-rated health was measured using one item asking participants to rate their overall physical health over the past two weeks. Responses were scored on a 5-point scale (1 = very poor, 5 = very good), with higher scores indicating better self-perceived health status.

#### 2.2.3 Mediating variables

The intention to use TCM services was evaluated using a 3-item scale designed to capture participants’ willingness to adopt TCM as a primary preventive measure, prioritize TCM institutions when seeking treatment, and adhere to prescribed therapies. Responses were rated on a 5-point Likert scale (1 = strongly disagree, 5 = strongly agree), with higher scores signifying a stronger inclination to use TCM services. The scale demonstrated robust internal consistency, achieving a Cronbach’s *α* coefficient of 0.911.

Loyalty was conceptualized across two dimensions, namely consultation willingness and recommendation intention, and operationalized using four items. Participants rated their agreement on a 5-point Likert scale, where higher aggregate scores reflected greater brand loyalty toward TCM services. With a Cronbach’s *α* of 0.951, the loyalty scale exhibited excellent reliability.

### 2.3 Explanatory variable

As shown in Table 1, the dependent variable Y (1 = use, 0 = no use) is the utilization of TCM serviece. Based on Anderson’s health behavior theoretical model, an independent variable (X) analysis framework composed of predisposing, enabling and need factors was constructed.

**Table 1:** Classification and definition of independent variables.

Factor	Variable	Definition
Predisposing factor		
Demographic characteristics	Age	Quantitative variable
	Sex	1 = Male
		2 = Female
	Marital status	1 = Unmarried
		2 = Married
		3 = Others
Occupational characteristics	Type of hospital	1 = General Hospital
		2 = TCM Hospital
		3 = Specialized Hospital
		4 = Primary Care Facilities
		5 = Others
	Occupation	1 = Clinician
		2 = Nurse
		3 = Medical technician
		4 = Others
	Officially budgeted post	1 = Yes
		2 = No
	Working years	Quantitative variable
Health cognition	Perceived value	Quantitative variable
	Satisfaction	Quantitative variable
Enabling factor		
Socioeconomic resources	Professional title	1 = Primary
		2 = Intermediate
		3 = Deputy Senior
		4 = Senior
		5 = Others
	Education	1 = Junior College and below
		2 = Bachelor
		3 = Master and above
Service accessibility	Accessibility	Quantitative variable
Need factor		
Objective health need	Chronic condition	1 = Yes
		2 = No
	Physical pain or discomfort	Quantitative variable
Subjective health need	Sleep quality	Quantitative variable
	Self-rated health	Quantitative variable

### 2.4 Data analysis

Data analyses were performed using Stata 17 and SPSS 27. Descriptive and univariate analyses were conducted to summarize the characteristics of healthcare professionals, with chi-square tests and t-tests applied for intergroup comparisons. Additionally, their TCM service utilization modalities and the TCM utilization rate among individuals with chronic diseases were analyzed separately. Finally, based on the Andersen Behavioral Model, a stepwise binary logistic regression analysis was employed to examine the effects of various factors on TCM service utilization. Multicollinearity in the multivariable logistic regression model was assessed using the variance inflation factor (VIF). Additionally, Model 4 of the SPSS macro PROCESS was utilized to examine the mediating roles of intention to use TCM and loyalty. A *p*-value less than 0.05 was considered statistically significant.

## 3 Results

### 3.1 Descriptive statistics

As shown in Table 2, a total of 1,483 respondents were included in the study. 80.11% of the respondents were female. The mean age was 35.71 ± 9.47 years. 69.18% of the participants had attained a bachelor’s degree. 72.62% were married. 48.35% held a primary professional title, and 33.04% held an intermediate professional title. The mean score for perceived value was 44.74 ± 9.77, and the mean score for satisfaction was 15.13 ± 3.27. Except for sex, hospital level, sleep quality, and self-rated health, significant differences in TCM service utilization were observed across other variables.

**Table 2:** Results of univariate analysis of TCM service utilization [*n* (%), Mean ± SD].

Variables	Total (*n* = 1483)	No use (*n* = 755)	Used (*n* = 728)	χ^2^, *t*	*P*
Sex					
Male	295 (19.89)	156 (20.66)	139 (19.09)	0.573	0.449
Female	1,188 (80.11)	599 (79.34)	589 (80.91)		
Age (years)	35.71 ± 9.47	34.33 ± 9.02	37.15 ± 9.72	−5.792	<0.001
Marital status					
Unmarried	380 (25.62)	224 (29.67)	156 (21.43)	13.552	0.001
Married	1,077 (72.62)	517 (68.48)	560 (76.92)		
Others	26 (1.75)	14 (1.85)	12 (1.65)		
Type of hospital					
General hospital	986 (66.49)	530 (70.20)	456 (62.64)	29.921	<0.001
TCM hospital	157 (10.59)	48 (6.36)	109 (14.97)		
Specialized hospital	14 (0.94)	7 (0.93)	7 (0.96)		
Primary care facilities	314 (21.17)	165 (21.85)	149 (20.47)		
Others	12 (0.81)	5 (0.66)	7 (0.96)		
Hospital Level					
Tertiary hospital	205 (13.82)	108 (14.30)	97 (13.32)	3.938	0.268
Secondary hospital	489 (32.97)	231 (30.60)	258 (35.44)		
Primary hospital	667 (44.98)	352 (46.42)	315 (43.27)		
Other	122 (8.23)	64 (8.48)	58 (7.97)		
Occupation					
Clinician	347 (23.40)	146 (19.34)	201 (27.61)	23.328	<0.001
Nurse	870 (58.66)	485 (64.24)	385 (52.88)		
Medical technician	176 (11.87)	88 (11.66)	88 (12.09)		
Others	90 (6.07)	36 (4.77)	54 (7.42)		
Officially budgeted post					
Yes	524 (35.33)	212 (28.08)	312 (42.86)	35.423	<0.001
No	959 (64.67)	543 (71.92)	416 (57.14)		
Working years	12.36 ± 9.93	10.92 ± 9.23	13.86 ± 10.40	−5.754	<0.001
Perceived value	44.74 ± 9.77	42.87 ± 9.38	46.69 ± 9.79	−7.667	<0.001
Satisfaction	15.13 ± 3.27	14.40 ± 3.11	15.89 ± 3.26	−9.016	<0.001
Professional title					
Primary	717 (48.35)	408 (54.04)	309 (42.45)	27.820	<0.001
Intermediate	490 (33.04)	229 (30.33)	261 (35.85)		
Senior	210 (14.16)				
Others	66 (4.45)	37 (4.90)	29 (3.98)		
Education					
Junior college and below	314 (21.17)	170 (22.52)	144 (19.78)	15.141	0.001
Bachelor	1,026 (69.18)	534 (70.73)	492 (67.58)		
Master and above	143 (9.64)	51 (6.75)	92 (12.64)		
Accessibility	11.41 ± 2.15	11.21 ± 2.23	11.62 ± 2.04	−3.658	<0.001
Chronic condition					
Yes	563 (37.96)	237 (31.39)	326 (44.78)	28.213	<0.001
No	920 (62.04)	518 (68.61)	402 (55.22)		
Physical pain or discomfort	2.65 ± 1.04	2.56 ± 1.04	2.75 ± 1.04	−3.540	<0.001
Sleep quality	2.80 ± 0.87	2.80 ± 0.86	2.80 ± 0.87	0.042	0.967
Self-rated health	3.20 ± 0.70	3.21 ± 0.71	3.18 ± 0.69	0.764	0.445

### 3.2 Current status of TCM service utilization by healthcare professionals

The distribution of TCM modalities shown in Table 3 provides contextual background for interpreting the determinants and mechanisms of overall TCM service utilization. In the past 12 months, 728 (49.09%) had used TCM treatment services, 755 (50.91%) had not used TCM treatment services. Among those who had used TCM treatment services, 73.63% of the healthcare professionals used massage and manipulation, 49.31% used TCM processing technology, and 42.72% used TCM external therapies.

**Table 3:** TCM services utilization modalities (*n* = 728).

Items of service	*n*	%
Massage and manipulation (manual techniques)	536	73.63
Chinese medicinal processing techniques (raw Chinese herbs, prepared slices, patent Chinese medicines, etc.)	359	49.31
Traditional Chinese external therapies (cupping, guasha, herbal baths, poultices, etc.)	311	42.72
Acupuncture (including needle embedding, acupoint injection)	257	35.30
Internal administration of Chinese medicine (herbal atomization inhalation, medicinal wines, tea infusions, medicinal diets, etc.)	255	35.03
Health Guidance	227	31.18
Moxibustion	189	25.96
Others	123	16.90
Modern assistive devices (magnetic therapy, electrotherapy, etc.)	86	11.81
Surgery (orthopedic, ophthalmic, proctologic, etc.)	13	1.79

### 3.3 TCM services utilization in individuals with chronic disease

Among the participants, 563 individuals were diagnosed with chronic diseases, accounting for 37.96% of the total sample. Within this subgroup, TCM service utilization rates varied by condition. Kidney disease (71.43%), musculoskeletal disease (60.49%), hypertension (58.59%), and hyperlipidemia (55.95%) all exceeding 55%. Additionally, the utilization rate was 51.72% for cardiovascular diseases and 51.22% for diabetes (Table 4).

**Table 4:** TCM service utilization in chronic patients (*n* = 563).

Chronic condition	*n*	Use TCM service	%
Kidney disease	7	5	71.43
Musculoskeletal disease	329	199	60.49
Hypertension	99	58	58.59
Hyperlipidemia	84	47	55.95
Cardiovascular diseases	29	15	51.72
Diabetes	41	21	51.22
Brain injury	2	1	50.00
Cerebrovascular diseases	9	4	44.44
Others	157	89	56.69

The sum of disease-specific counts (757) exceeds the number of individuals with any chronic disease (563) because participants could report multiple conditions.

### 3.4 Multivariable logistic regression and mediation analyses

Severe multicollinearity was observed between age and working years (VIF > 10). Given their high correlation and the theoretical priority of age as a core demographic variable, working years was excluded from the model. The VIF values for all remaining variables ranged from 1.05 to 3.89, well below the threshold of 5, suggesting no severe multicollinearity in the final model.

As shown in Table 5, four models were constructed using stepwise binary logistic regression to examine factors associated with TCM service utilization among healthcare professionals, based on the Andersen Behavioral Model. Model 1, which included all predisposing factors except satisfaction, found that older age (OR = 1.020, *p* < 0.05), female (OR = 1.820, *p* < 0.001), TCM hospital employment (OR = 2.240, *p* < 0.001), and higher perceived value (OR = 1.037, *p* < 0.001) were significantly associated with increased TCM services utilization, whereas nurses (OR = 0.676, *p* < 0.05) and individuals without an official budgeted post (OR = 0.648, *p* < 0.01) were less likely to use TCM services compared with their counterparts. Model 2 included the full set of predisposing factors. In this model, higher satisfaction was significantly associated with increased TCM services utilization (OR = 1.149, *p* < 0.001), while perceived value became non-significant after mutual adjustment (*p* > 0.05). Model 3 further adjusted for enabling factors, no enabling factor showed an independent association with TCM use (all *p* > 0.05). Model 4, the fully adjusted model including need factors, showed that for each 1-point increase in physical pain or discomfort, the odds of TCM service utilization increased by 31.3% (OR = 1.313, *p* < 0.001). Compared with participants with chronic conditions, those without chronic conditions were less likely to use TCM services (OR = 0.730, *p* < 0.05).

**Table 5:** Results of binary logistic regression models for TCM service utilization.

Variable	Model 1		Model 2		Model 3		Model 4	
	OR (95%CI)	*P*	OR (95%CI)	*P*	OR (95%CI)	*P*	OR (95%CI)	*P*
Predisposing factor								
Sex								
Male	ref		ref		ref		ref	
Female	1.820 (1.320–2.510)	<0.001	1.902 (1.374–2.631)	<0.001	1.865 (1.345–2.586)	<0.001	1.814 (1.301–2.530)	<0.001
Age (years)	1.020 (1.003–1.038)	0.024	1.017 (0.999–1.034)	0.063	1.017 (0.996–1.039)	0.104	1.011 (0.990–1.034)	0.303
Marital status								
Unmarried	ref		ref		ref		ref	
Married	1.066 (0.790–1.438)	0.675	1.094 (0.810–1.479)	0.558	1.058 (0.766–1.462)	0.730	1.086 (0.783–1.507)	0.621
Others	0.709 (0.298–1.689)	0.438	0.717 (0.302–1.701)	0.451	0.716 (0.302–1.701)	0.449	0.685 (0.283–1.660)	0.402
Type of hospital								
General hospital	ref		ref		ref		ref	
TCM hospital	2.240 (1.483–3.384)	<0.001	2.155 (1.423–3.266)	<0.001	2.179 (1.427–3.326)	<0.001	2.147 (1.397–3.298)	<0.001
specialized hospital	1.019 (0.333–3.120)	0.973	0.897 (0.288–2.789)	0.850	0.796 (0.255–2.491)	0.695	0.868 (0.269–2.801)	0.813
primary care facilities	0.835 (0.441–1.578)	0.578	0.808 (0.427–1.531)	0.513	0.818 (0.431–1.554)	0.540	0.844 (0.441–1.617)	0.609
Others	1.608 (0.470–5.504)	0.449	1.534 (0.442–5.327)	0.501	1.599 (0.456–5.604)	0.464	1.836 (0.510–6.608)	0.353
Hospital level								
Tertiary hospital	ref		ref		ref		ref	
Secondary hospital	1.180 (0.627–2.221)	0.607	1.294 (0.686–2.441)	0.426	1.318 (0.698–2.490)	0.395	1.230 (0.646–2.344)	0.529
Primary hospital	0.804 (0.422–1.533)	0.508	0.841 (0.441–1.607)	0.601	0.769 (0.401–1.477)	0.431	0.729 (0.377–1.412)	0.349
Other	0.960 (0.599–1.539)	0.866	0.955 (0.594–1.536)	0.850	0.971 (0.604–1.560)	0.902	1.014 (0.627–1.640)	0.955
Occupation								
Clinician	ref		ref		ref		ref	
Nurse	0.676 (0.488–0.935)	0.018	0.671 (0.483–0.931)	0.017	0.781 (0.541–1.128)	0.188	0.752 (0.517–1.092)	0.134
Medical technician	0.709 (0.481–1.046)	0.083	0.729 (0.493–1.077)	0.112	0.807 (0.538–1.210)	0.299	0.833 (0.551–1.258)	0.384
Others	1.088 (0.656–1.806)	0.743	1.122 (0.674–1.867)	0.659	1.214 (0.717–2.053)	0.470	1.146 (0.673–1.949)	0.616
Officially budgeted post								
Yes	ref		ref		ref		ref	
No	0.648 (0.484–0.869)	0.004	0.640 (0.477–0.860)	0.003	0.668 (0.493–0.905)	0.009	0.690 (0.507–0.939)	0.018
Perceived value	1.037 (1.025–1.049)	<0.001	0.997 (0.976–1.019)	0.816	0.997 (0.975–1.019)	0.782	1.005 (0.983–1.028)	0.665
Satisfaction			1.149 (1.076–1.226)	<0.001	1.150 (1.078–1.228)	<0.001	1.142 (1.069–1.219)	<0.001
Enabling factor								
Professional title								
Primary					ref		ref	
Intermediate					1.057 (0.781–1.430)	0.719	1.044 (0.769–1.418)	0.780
Senior					1.076 (0.656–1.764)	0.772	1.158 (0.701–1.915)	0.567
Others					1.003 (0.569–1.770)	0.991	1.068 (0.601–1.896)	0.823
Education								
Junior College and below					ref		ref	
Bachelor					1.060 (0.787–1.427)	0.701	1.034 (0.764–1.397)	0.830
Master and above					1.657 (0.962–2.854)	0.069	1.660 (0.955–2.886)	0.073
Accessibility					0.989 (0.934–1.047)	0.700	1.020 (0.961–1.082)	0.522
Need factor								
Chronic condition								
Yes							ref	
No							0.730 (0.568–0.938)	0.014
Physical pain or discomfort							1.313 (1.143–1.509)	<0.001
Sleep quality							1.047 (0.892–1.228)	0.574
Self-rated health							0.950 (0.780–1.157)	0.612
Constant	0.095 (0.032–0.287)	<0.001	0.069 (0.023–0.213)	<0.001	0.064 (0.017–0.238)	<0.001	0.028 (0.006–0.141)	<0.001

**Figure 1:**
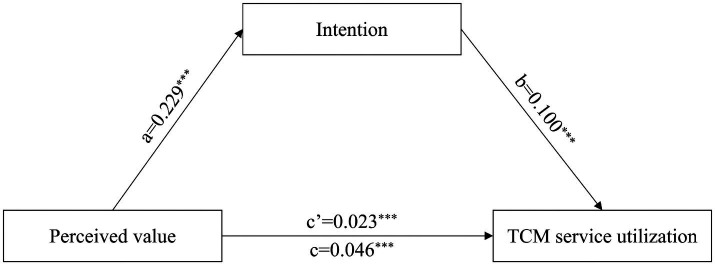
The mediation model between perceived value and TCM service utilization. ^***^p < 0.001.

**Figure 2:**
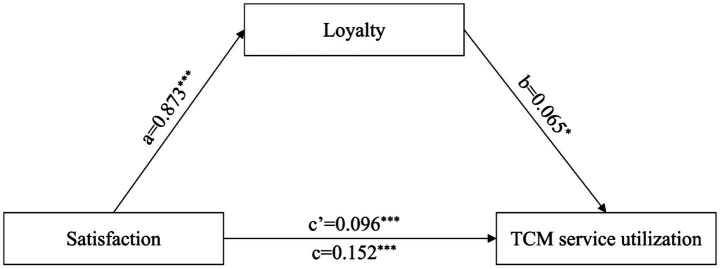
The mediation model between satisfaction and TCM service utilization. ^*^p < 0.05, ^***^p < 0.001.

Table 6 and Figures 1, 2 show the mediating role of intention to use TCM and loyalty. Intention to use TCM significantly mediated the association between perceived value and TCM service utilization, with 50.00% of the association (*p* < 0.05). Loyalty significantly mediated the association between satisfaction and TCM service utilization, with 36.84% of the association (*p* < 0.05).

**Table 6:** Intention to use TCM and loyalty in the mediation effect analysis.

Path	Effect type	Effect	Boot SE	Boot LLCI	Boot ULCI	Mediation effect ratio
Perceived value→Intention→TCM service utilizatio	Direct effect	0.023	0.010	0.003	0.044	50.00%
	Indirect effect	0.023	0.009	0.007	0.041	
	Total effect	0.046	0.006	0.034	0.058	
Satisfaction→Loyalty→TCM service utilization	Direct effect	0.096	0.029	0.039	0.152	36.84%
	Indirect effect	0.056	0.024	0.009	0.101	
	Total effect	0.152	0.021	0.117	0.187	

Control varilables: sex, age, education, marital status, type of hospital, occupation, professional title, officially budgeted post, working years, chronic condition.

## 4 Discussion

Based on the Andersen Model, this study explored the influencing factors of TCM service utilization among healthcare professionals and examined the mediating roles of TCM service utilization intention and loyalty. The results showed that the utilization rate of TCM services among healthcare professionals in the past 12 months was less than half (49.09%), with massage and manipulation being the main forms of utilization. Additionally, the utilization rate was significantly higher among healthcare professionals with chronic condition, especially those with kidney diseases and musculoskeletal disorders. Meanwhile, age, sex, type of hospital, officially budgeted post, occupation, perceived value, satisfaction, chronic condition, and physical pain or discomfort had a significant positive impact on TCM service utilization among healthcare professionals. Furthermore, the mediating pathways of perceived value → TCM service utilization intention → TCM service utilization and satisfaction → loyalty → TCM service utilization were confirmed to be valid.

### 4.1 Current status of TCM service utilization among medical staff

This study found that the utilization rate of TCM services among healthcare professionals was 49.09%, which was less than half. This rate was lower than that of the general population (20), possibly because healthcare professionals rely more on evidence-based evidence when selecting medical services and require more comprehensive professional knowledge to accept TCM. Meanwhile, healthcare professionals often face heavy workloads, busy clinical schedules, and limited time for self-care (21), which may restrict their access to and use of TCM services. At the institutional level, differences in the availability of TCM resources, service settings, and practice environments may also create barriers to utilization. Existing systematic reviews have indicated that the clinical practice environment and local health service resources are closely associated with the utilization of traditional and complementary medicine (22). Therefore, the relatively low utilization rate among healthcare professionals is not only due to evidence-based preferences but also jointly shaped by time constraints, occupational pressures, and institutional support for TCM services.

In terms of treatment modalities, massage and manual techniques had the highest utilization rate (73.63%). The reason might be that massage and manual techniques take effect quickly, have no drug side effects, and are easy to operate, which better meet the needs of healthcare professionals to efficiently alleviate local discomfort (23). Given these accessible and user-friendly features, such a prevalent use of massage and manipulation also helps explain why cognitive and attitudinal factors, including perceived value, satisfaction, utilization intention, and loyalty, showed significant predictive effects on overall TCM service utilization. These characteristics strengthen the role of psychological perceptions in driving actual utilization behavior.

Traditional Chinese herb has unique advantages in chronic disease management (24), while external TCM therapies excel in relieving local pain (25, 26). Therefore, healthcare professionals are also inclined to select these treatment modalities, consistent with Guo et al. (27) study on outpatients in tertiary TCM hospitals. Their study found that traditional Chinese herb, acupuncture, and moxibustion therapies were the most commonly used in outpatient settings. Notably, the overall utilization rate in Guo et al. (27) study (68.23%) was higher than ours. Such a difference might be due to varying availability of TCM resources across sampling regions; specifically, full-time TCM practitioners are stationed at primary medical institutions in certain areas, which reduces the utilization costs of traditional Chinese medicine and external TCM therapies.

Among healthcare professionals, 37.96% suffered from chronic diseases. Among these individuals with chronic conditions, those diagnosed with kidney diseases, musculoskeletal disorders, hypertension, and hyperlipidemia showed a higher acceptance of TCM services. This result is highly consistent with the advantageous fields of TCM treatment. TCM has well-established clinical applications in the management of chronic kidney diseases and musculoskeletal disorders (28, 29). These types of conditions are more suitable for intervention through a combined approach of TCM herbal therapy and adjuvant external TCM treatments. Notably, the high utilization rates of TCM herbal processing techniques and external TCM therapies observed in this study further confirm healthcare professionals’ recognition of the synergistic effects of multi-modal TCM treatment for chronic diseases. For conditions such as hypertension and diabetes healthcare professionals predominantly adopt a Western medicine-based, TCM-adjuvant model (30, 31). This is because these diseases require long-term standardized Western medical management to control blood pressure and blood glucose, respectively. Consequently, although the utilization rate of TCM for these conditions is slightly lower, it still remains close to 50%. Despite the promising potential of TCM demonstrated in chronic disease management and clinical practice, large-scale, high-quality clinical evidence supporting its efficacy remains insufficient. Future studies should prioritize strengthening TCM technical training. They should also expand sample coverage and conduct in-depth investigations into the mechanisms underlying the application of TCM in chronic disease management. These efforts will provide more robust evidence for the clinical promotion of TCM.

### 4.2 Influencing factors of TCM service utilization

This study found that age, sex and chronic disease status had significant effects on the utilization of TCM services among healthcare professionals. Marital status showed no significant association with TCM service utilization. This finding is fully consistent with the earlier result that the utilization rate of TCM among healthcare professionals with chronic diseases exceeded 50% and reached 70% for some specific disease types. Healthcare professionals with chronic diseases have more urgent personal health needs. They are also more likely to perceive the value of TCM in chronic disease management. These factors contribute to their higher probability of using TCM services. The impact of age may be reflected in the higher prevalence of chronic diseases among older healthcare professionals. This indirectly promotes the utilization of TCM. This result aligns with the research findings of Cao et al. (32). Their study indicated that the proportion of patients with chronic diseases who used TCM treatment was 49.07%. It also pointed out that factors such as age, type of chronic disease and comorbidity status significantly affected the use of TCM treatment. As for the non-significant impact of marital status, it may be because healthcare professionals’ decisions on TCM utilization rely more on professional judgment rather than family-related factors.

In addition, type of hospital and physical pain or discomfort were significantly associated with TCM service utilization. Healthcare professionals working in TCM hospitals showed higher utilization rates, which may be attributed to greater exposure to TCM practice, easier access to TCM resources, and a more supportive clinical environment. This observation is supported by Ren et al. (33), who demonstrated that those with a convenient access to health service demonstrated an observed higher propensity to utilize TCM services. Furthermore, physical pain or discomfort significantly increased the likelihood of TCM use. TCM modalities such as massage are convenient and effective in relieving local discomfort, which matches the needs of healthcare professionals with occupational fatigue. A study by Ding et al. has shown that Chinese massage is effective in relieving chronic pain and is widely used as a nonpharmacological therapeutic approach (34). This may partly explain why physical discomfort serves as an important driving factor for TCM utilization in this population.

Perceived value and satisfaction had a significant positive impact on the utilization of TCM services. This finding echoes the conclusion of Zeng et al. (35) that the higher an individual’s perceived value of TCM the stronger their willingness to use it. The present study further confirms that perceived value is also a key variable among the need factors in the healthcare professional group. As a professional group healthcare professionals make value judgments on TCM services more based on professional dimensions such as efficacy and safety. If they perceive that TCM can meet their own health needs they are more likely to translate this perception into actual utilization. Satisfaction reflects healthcare professionals’ overall evaluation of and attitudes toward TCM services, rather than merely an assessment of past personal usage experiences. Positive attitudes toward TCM services strengthen healthcare professionals’ subsequent TCM utilization behavior.

### 4.3 Deepened analysis of the mediating mechanism

The mediating effect of utilization intention was significant. That is, perceived value can further promote the utilization of TCM services only by enhancing utilization intention. For healthcare professionals, perceived value is a judgment at the cognitive level, while utilization intention is a choice at the behavioral tendency level. Positive cognition alone is not sufficient to directly translate into behavior. It is necessary to first form a tendency to be willing to use TCM, and then implement it into actual utilization. This is consistent with the health behavior logic of cognition → attitude → behavior. This pathway is consistent with the research findings of Xia et al. (36) in the general resident group. However, the mediating effect of utilization intention in this study is stronger. This may be because the transformation cost from intention to behavior is lower for healthcare professionals.

The mediating effect of loyalty was confirmed. Satisfaction promotes the utilization of TCM services by enhancing loyalty. Satisfaction is an evaluation of past service usage experiences. When healthcare professionals have high satisfaction with their previous TCM service experiences they will develop loyalty that includes willingness to use TCM again and recommend it to others. This loyalty significantly reduces hesitation in their subsequent decisions regarding TCM utilization and thus improves the actual utilization rate. Both pathways indicate that cognition or experience needs to be transformed into utilization behavior through intermediate variables. This confirms the necessity of supplementing mediating mechanisms in this study.

### 4.4 Strengths and limitations

This study has several strengths. First, it focuses on healthcare professionals, a group that has been less frequently examined in studies of TCM utilization. As both providers and potential users of healthcare, their perspectives may offer additional insight into how and why TCM services are used. Second, we applied the Andersen Behavioral Model to organize the analysis, considering predisposing, enabling, and need factors together. This allowed us to look at TCM utilization from a more integrated perspective, rather than examining individual factors in isolation. Finally, we further explored possible pathways linking these factors to utilization by including intention and loyalty in the analysis.

Several limitations warrant acknowledgment. Primarily, the sample was geographically constrained to specific healthcare institutions in Nantong, restricting the ability to extrapolate findings to broader contexts. Furthermore, reliance on self-reported questionnaire data introduced the risk of response bias, as subjective accounts may not always align with actual behavioral patterns. Additionally, the exclusive focus on medical professionals who possess specialized healthcare knowledge limits the transferability of these results to the general public, whose perceptions of TCM likely differ substantially. Finally, our analysis treated TCM service utilization as a binary variable without distinguishing between different modalities. Perceived value, satisfaction, and the mediating roles of intention and loyalty may differ substantially across service types.

Future studies could take a comparative approach—including not just healthcare professionals but also other demographic groups. This would help clarify how TCM utilization and perceived value differ across populations. Also, with larger and more balanced samples, researchers could examine whether the mediating pathways we identified hold consistently across different TCM modalities, or whether they vary depending on the type of service used.

## 5 Conclusion

In conclusion, our study indicated that TCM service utilization among healthcare professionals still has scope for improvement. Among the predisposing factors, the factors affecting the TCM service utilization rate are sex, officially budgeted post, perceived value and satisfaction. Among the need factors, the factors influencing the TCM service utilization rate are chronic condition and physical pain or discomfort. Perceived value indirectly promotes TCM service utilization via utilization intention, and satisfaction via loyalty. This dual mediating mechanism confirms the necessity of converting cognition and experience into behavior. Therefore, to increase the utilization rate of TCM services among healthcare professionals, targeted measures should be taken based on the chronic disease status and educational level of different groups of healthcare professionals, starting with TCM training in medical institutions, enhancing their professional recognition through TCM knowledge dissemination by professional TCM personnel, and focusing on the cultivation of utilization intention and loyalty. In addition, the state should also pay attention to the promotion of TCM services among healthcare professionals and increase investment in TCM resource allocation and evidence-based research.

## Data Availability

The original contributions presented in the study are included in the article/Supplementary material, further inquiries can be directed to the corresponding author/s.
