# Supplementary material for: The prevalence, determinants, and mechanisms of traditional Chinese medicine service utilization among healthcare professionals: a study based on the Andersen Behavioral Model
Source: Front Public Health. 2026 May 13;14:1827392. doi: 10.3389/fpubh.2026.1827392 (PMC13212355; doi:10.3389/fpubh.2026.1827392)
Supplement: Supplementary file 1 [file Data_Sheet_1.PDF]

# Questionnaire on Traditional Chinese Medicine (TCM) Service Utilization

| **Part I Basic Personal Information** | | |
|---|---|---|
| A1 | Sex | 1 Male<br>2 Female |
| A2 | Date of Birth | ____________ Year<br>____________ Month |
| A3 | Education | 1 Junior College and below<br>2 Bachelor<br>3 Master and above |
| A4 | Marital status | 1 Unmarried<br>2 Married<br>3 Others |
| A5 | Type of hospital | 1 General Hospital 4 Primary Care Facilities<br>2 TCM Hospital 5 Others<br>3 Specialized Hospital |
| A6 | Hospital Level | 1 Tertiary Hospital 3 Primary hospital<br>2 Secondary Hospital 4 Other |
| A7 | Hospital Nature | 1 Public hospital<br>2 Private hospital<br>3 Others |
| A8 | Occupation | 1 Clinician 3 Medical technician<br>2 Nurse 4 Others |
| A9 | Professional title | 1 Primary 3 Senior<br>2 Intermediate 4 Others |
| A10 | Officially budgeted post | 1 Yes<br>2 No |
| A11 | Working years | _____________ Years |
| A12 | Compared with others around you, how would you rate your household income over the past year? | 1 Very low 4 Relatively high<br>2 Relatively low 5 Very high<br>3 Moderate |

| | | |
|---|---|---|
| **Part II Physical Health Status** | | |
| B1 | Over the past year, how would you rate your overall sleep quality? | 1 Very poor<br>2 Poor<br>3 Fair<br>4 Good<br>5 Very good |
| B2 | Over the past year, how often have you been bothered by pain or physical discomfort? | 1 Never<br>2 Occasionally<br>3 Sometimes<br>4 Often<br>5 Very often |
| B3 | Have you ever been diagnosed with any of the following chronic conditions by a physician? (Multiple choice) | 0 No<br>1 Diabetes<br>2 Hypertension<br>3 Hyperlipidemia<br>4 Cardiovascular Diseases<br>5 Cerebrovascular Diseases<br>6 Brain injury<br>7 Kidney Disease<br>8 Musculoskeletal Disease<br>9 Others |
| B4 | Over the past year, what was your participation in health examinations? | 1 Did not undergo any health examination<br>2 Self-paid health examination<br>3 Regular community-organized health examinations<br>4 Regular workplace-organized health examinations |
| B5 | Over the past two weeks, how would you rate your overall physical health? | 1 Very poor<br>2 Poor<br>3 Fair<br>4 Good<br>5 Very good |
| **Part III Accessibility of TCM Services** | | |
| C1 | How long does it take to travel from your home to the nearest TCM institution you are aware of? | 1 >1 hour<br>2 45 – 60 minutes<br>3 30 – 45 minutes<br>4 15 – 30 minutes<br>5 <15 minutes |
| C2 | Do you consider the distance from your home to the nearest TCM institution to be appropriate? | 1 Very inappropriate<br>2 Inappropriate<br>3 Slightly inappropriate<br>4 Appropriate<br>5 Very appropriate |
| C3 | Do you think your and your family's healthcare needs can be met by nearby TCM institutions? | 1 Very unmet<br>2 Unmet<br>3 Slightly unmet<br>4 Met<br>5 Fully met |

| **Part IV Perceived Value of TCM Services** | | |
|---|---|---|
| **Economic Value** | | |
| D1 | Overall, the price of TCM services is reasonable | 1 Strongly disagree<br>2 Disagree<br>3 Neutral<br>4 Agree<br>5 Strongly agree |
| D2 | Overall, the price of TCM medicines is reasonable. | 1 Strongly disagree<br>2 Disagree<br>3 Neutral<br>4 Agree<br>5 Strongly agree |
| D3 | Overall, TCM services offer good value for money. | 1 Strongly disagree<br>2 Disagree<br>3 Neutral<br>4 Agree<br>5 Strongly agree |
| **Functional Value** | | |
| D4 | My condition improved after the most recent TCM treatment. | 1 Strongly disagree<br>2 Disagree<br>3 Neutral<br>4 Agree<br>5 Strongly agree |
| D5 | I was satisfied with the treatment process during my most recent TCM use. | 1 Strongly disagree<br>2 Disagree<br>3 Neutral<br>4 Agree<br>5 Strongly agree |
| D6 | The cost of my most recent TCM treatment was reasonable. | 1 Strongly disagree<br>2 Disagree<br>3 Neutral<br>4 Agree<br>5 Strongly agree |
| **Trust Value** | | |
| D7 | This institution has strong TCM expertise. | 1 Strongly disagree<br>2 Disagree<br>3 Neutral<br>4 Agree<br>5 Strongly agree |
| D8 | This institution has a high reputation in TCM. | 1 Strongly disagree<br>2 Disagree<br>3 Neutral<br>4 Agree<br>5 Strongly agree |
| D9 | This institution is capable of providing high-quality TCM services. | 1 Strongly disagree<br>2 Disagree<br>3 Neutral<br>4 Agree<br>5 Strongly agree |

| | | |
|---|---|---|
| D10 | This institution has a good reputation in TCM diagnosis and treatment. | 1 Strongly disagree<br>2 Disagree<br>3 Neutral<br>4 Agree<br>5 Strongly agree |
| D11 | This institution actively responds to patients' needs. | 1 Strongly disagree<br>2 Disagree<br>3 Neutral<br>4 Agree<br>5 Strongly agree |
| D12 | I would recommend this institution's TCM services to my patients when needed. | 1 Strongly disagree<br>2 Disagree<br>3 Neutral<br>4 Agree<br>5 Strongly agree |
| **Part V Satisfaction with TCM Services** | | |
| E1 | Overall, TCM is effective in prevention and health promotion. | 1 Strongly disagree<br>2 Disagree<br>3 Neutral<br>4 Agree<br>5 Strongly agree |
| E2 | Overall, TCM is effective in treating chronic or complex diseases. | 1 Strongly disagree<br>2 Disagree<br>3 Neutral<br>4 Agree<br>5 Strongly agree |
| E3 | Overall, I am satisfied with the service attitude of TCM practitioners. | 1 Strongly disagree<br>2 Disagree<br>3 Neutral<br>4 Agree<br>5 Strongly agree |
| E4 | Compared with Western medicine, TCM provides better treatment outcomes. | 1 Strongly disagree<br>2 Disagree<br>3 Neutral<br>4 Agree<br>5 Strongly agree |
| **Part VI Loyalty to TCM Services** | | |
| F1 | If I become ill, I would prioritize TCM services. | 1 Strongly disagree<br>2 Disagree<br>3 Neutral<br>4 Agree<br>5 Strongly agree |
| F2 | I would recommend TCM services to colleagues and friends. | 1 Strongly disagree<br>2 Disagree<br>3 Neutral<br>4 Agree<br>5 Strongly agree |
| F3 | I intend to continue using TCM services in the long term. | 1 Strongly disagree<br>2 Disagree<br>3 Neutral<br>4 Agree<br>5 Strongly agree |

| | | |
|---|---|---|
| F4 | I will continue to follow TCM-related health information provided by hospitals. | 1 Strongly disagree<br>2 Disagree<br>3 Neutral<br>4 Agree<br>5 Strongly agree |
| **Part VII Intention to Use TCM Services** | | |
| G1 | I am willing to use TCM as a primary method for prevention and health care. | 1 Strongly disagree<br>2 Disagree<br>3 Neutral<br>4 Agree<br>5 Strongly agree |
| G2 | When I become ill, I am willing to first seek care from TCM institutions. | 1 Strongly disagree<br>2 Disagree<br>3 Neutral<br>4 Agree<br>5 Strongly agree |
| G3 | After my most recent TCM treatment, I followed the doctor's advice for continued treatment. | 1 Strongly disagree<br>2 Disagree<br>3 Neutral<br>4 Agree<br>5 Strongly agree |
| **Part VIII Utilization of TCM Services** | | |
| H1 | Have you used any TCM services in the past 12 months? | 0 No<br>1 Yes |
| H2 | What types of TCM services have you used in the past 12 months? (Multiple choice) | 1 Acupuncture (including needle embedding, acupoint injection)<br>2 Massage and Manipulation (manual techniques)<br>3 Moxibustion<br>4 Traditional Chinese External Therapies (cupping, guasha, herbal baths, poultices, etc.)<br>5 Chinese Medicinal Processing Techniques (raw Chinese herbs, prepared slices, patent Chinese medicines, etc.)<br>6 Internal Administration of Chinese Medicine (herbal atomization inhalation, medicinal wines, tea infusions, medicinal diets, etc.)<br>7 Modern Assistive Devices (magnetic therapy, electrotherapy, etc.)<br>8 Surgery (orthopedic, ophthalmic, proctologic, etc.)<br>9 Health Guidance<br>10 Others |
